# Peroxide-Free Titanium Dioxide Nanoparticle–Based Photocatalytic Bleaching: In Vitro Study on Bovine Teeth

**DOI:** 10.1155/bmri/9311501

**Published:** 2025-03-20

**Authors:** Tainah Oliveira Rifane, Isabel Silva Nascimento, Suely Cristina Aragão Veras Santos, Lucca Reis Mesquita, Diego Martins de Paula, Victor P. Feitosa

**Affiliations:** ^1^Department of Restorative Dentistry, Paulo Picanço School of Dentistry, Fortaleza, Ceará, Brazil; ^2^Department of Operative Dentistry, University of Iowa College of Dentistry, Iowa City, Iowa, USA

**Keywords:** bleaching, hydrogen peroxide, titanium dioxide, violet LED

## Abstract

**Objective:** This study is aimed at assessing the bleaching efficacy of titanium dioxide (TiO2) nanoparticle suspensions at different concentrations and exposure times for photocatalysis and evaluating their potential as a peroxide-free alternative to carbamide peroxide in dental bleaching.

**Materials and Methods:** Sixty bovine incisors were stained with black tea and treated with different bleaching protocols: (1) control (16% carbamide peroxide), (2) TiO_2_-50 wt% for 10 min (TiO_2_ 50/10), (3) TiO_2_-50 wt% for 50 min (TiO_2_ 50/50), (4) TiO_2_-10 wt% for 50 min (TiO2 10/50), and (5) TiO_2_-10 wt% for 10 min (TiO_2_ 10/10). The agents were exposed to UV light (395 nm) for photocatalysis before application. All treatments were applied daily for 2 h over 21 days. Color change (Δ*E*00) was measured using a digital spectrophotometer (VITA Easyshade V), and pH was assessed using pH strips.

**Results:** The data were analyzed using one-way ANOVA and Tukey's test. TiO_2_ 50/50 showed significantly superior bleaching effects compared to all other groups (*p* < 0.001). TiO_2_ 10/50 and TiO_2_ 10/10 demonstrated intermediate bleaching results, with no significant difference between them (*p* = 0.0875). The control and TiO_2_ 50/10 groups exhibited the lowest color variation (*p* = 0.102). All groups maintained a pH of 6 before and after 2 h of the at-home bleaching protocol.

**Conclusion:** TiO_2_ nanoparticle suspensions may be a viable peroxide-free alternative for dental bleaching, particularly at higher concentrations and longer photocatalysis exposure times.

## 1. Introduction

Dental bleaching is a widely practiced clinical procedure recognized for its conservative and straightforward nature. This process involves the removal of pigments deposited on the tooth structure through a nonspecific chemical oxidation reaction facilitated by bleaching gels [1, 2]. The two primary approaches to teeth bleaching are home whitening and in-office bleaching, which differ in peroxide concentration, formulation type, pH, and application methods. The effectiveness of bleaching treatments is influenced by various factors, with particular emphasis on the characteristics of the pigment and the interaction between the bleaching agent and the tooth structure [3–5].

The major strategy to provide tooth whitening is by using hydrogen peroxide (HP), released through the decomposition of carbamide peroxide (CP), acting as a precursor that generates reactive species, including free radicals such as the perhydroxyl anion and hydroxyl radical [1–4]. These radicals facilitate the breakdown of high molecular weight pigments containing unsaturated bonds or aromatic rings into smaller, more water-soluble components, which then diffuse into the tooth structure, resulting in color changes [1, 6]. The most common hypothesis for bleaching sensitivity is related to HP molecules easily diffusing through the dentin, the periodontal ligament, and the nerve endings of the pulp, potentially triggering inflammatory reactions and dentin hypersensitivity [1, 5]. The severity of these effects is influenced by factors such as HP concentration, application time, pH, and the composition of the bleaching agent. Higher concentrations are associated with increased dentin hypersensitivity and greater pulpal inflammation, phenomena frequently observed in some patients [7–9].

To mitigate the drawbacks associated with peroxide use, several investigations have focused on identifying alternative materials that effectively whiten teeth without inducing sensitivity or compromising enamel surface roughness, microhardness, and fracture resistance [10, 11]. Some studies have explored the combination of various chemical elements to reduce HP concentration while accelerating the oxidation reaction, utilizing compounds such as cobalt–tetraphenylporphyrin/graphene oxide nanocomposite [12], chitosan [13], nitrogen, and ozone [11] combined with TiO₂ [14]. Many of these substances do not significantly alter the physicochemical properties, morphology, or microhardness of enamel [14–18]. However, the reduced HP concentration is associated with diminished initial bleaching effectiveness and less pronounced long-term effects [15, 16, 19].

Titanium dioxide (TiO₂) is a semiconductor that absorbs photons through the process of photocatalysis, resulting in the excitation of electrons from the valence band (VB) to the conduction band (CB). This mechanism generates free radicals, such as hydroxyl radicals, which are essential for the degradation of pigment molecules. Research indicates that combining TiO₂ with HP significantly reduces the clinical time required for dental whitening procedures. When irradiated with violet light, a gel containing TiO₂ and 6% HP exhibits whitening efficacy superior to that of 6% HP alone, achieving results comparable to those obtained with 35% HP. Additionally, this method shows promise in minimizing cytotoxic damage and, consequently, bleaching sensitivity. TiO₂ is characterized by its high photocatalytic activity, chemical stability, and excellent biocompatibility [15–20].

Incorporating TiO₂ nanoparticles into bleaching gels may help mitigate certain negative aspects associated with HP [14, 15, 18, 21]. Although numerous investigations have integrated TiO₂ nanoparticles while reducing peroxide concentration, to our knowledge, no study has exclusively employed a photocatalytic approach using ultraviolet light (UV) with wavelengths in the violet range of approximately 385–440 nm without any peroxide. In a previous pilot study, we evaluated a suspension of TiO₂ in water, devoid of peroxide, which yielded promising bleaching results. This finding encouraged further research to determine the optimal concentration of TiO₂ nanoparticles and the best exposure time for photocatalysis to maximize bleaching potential [20–22].

Therefore, the present study is aimed at assessing the at-home dental bleaching efficacy of suspensions containing varying concentrations of TiO₂ nanoparticles and investigating the effects of different UV exposure times on photocatalysis, comparing the outcomes with those of a CP bleaching protocol. The null hypothesis was that there would be no significant difference between the concentrations/times of the tested bleaching agents and CP.

## 2. Material and Methods

### 2.1. Specimen Preparation

This study utilized 60 donated bovine incisors with intact crowns. The teeth were meticulously cleaned using periodontal curettes (Gracey 5–6, Millennium, São Paulo, Brazil) and polished with a rubber cup (Microdont, São Paulo, Brazil) at low speed, in conjunction with water and pumice. Following the cleaning process, the teeth were stored in distilled water for no longer than 1 month. All specimens were then sectioned at the cementoenamel junction (CEJ) using a diamond saw on a cutting machine (Isomet, Buehler, Lake Bluff, Illinois, United States) to remove the roots. To prevent dye penetration into the dentin of the pulp chamber, the root canals were sealed with wax. Subsequently, the teeth were immersed in a black tea infusion and stored at 37°C for 1 week to induce pigmentation. The black tea infusion was prepared using 250 mL of boiling water and 18 g of black tea powder (Dr. Oetker, São Paulo, Brazil) [22]. After the pigmentation period, all teeth were thoroughly rinsed under running water and stored. To ensure consistent and standardized coloration, teeth with very light or darker shades were excluded from the study, resulting in the selection of teeth with shades A3, B3, and A3.5 for standardization purposes. These were then randomly distributed among the different experimental groups.

### 2.2. TiO₂ Suspension Preparation and Bleaching Protocol

**Figure 1:**
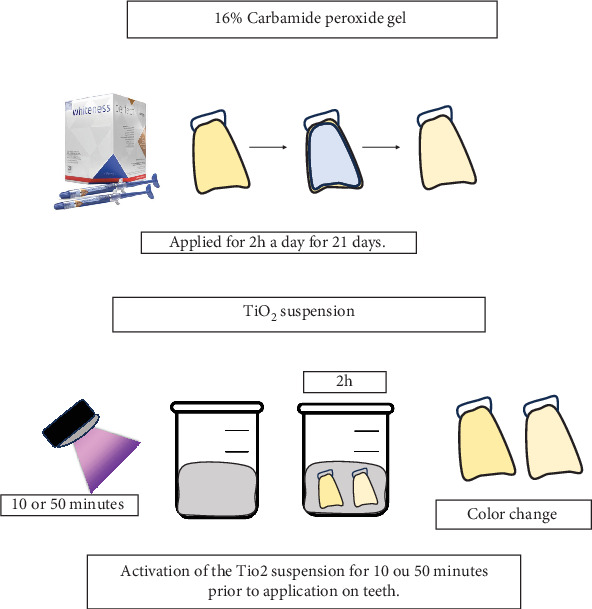
At-home bleaching protocols were used in the study with commercial and experimental bleaching agents with TiO_2_ in different concentrations and activation times for photocatalysis.

Suspensions containing TiO₂ nanoparticle powder (Sigma-Aldrich, St. Louis, United States) were prepared using distilled water at two different concentrations: 10 and 50 wt% [23]. Each suspension was made by mixing 1.5 mL of water with TiO₂ powder, which had an average particle size of 30 nm and predominantly consisted of anatase and rutile phases (50:50). The mixture was homogenized using a vortex for 30 s. After preparation, the suspensions were stored in light-protected tubes for subsequent photocatalysis and application to the teeth. Prior to application, the suspensions underwent a photocatalysis process involving exposure to UV (395 nm wavelength) with an exitance power of 2 W; specimens were kept 3 mm from the UV lamp, achieving a final irradiance of 200 mW/cm^2^ (2CPS, Opus, São Paulo, Brazil). Two different exposure times were used: 10 and 50 min [14]. After photocatalysis, the suspensions were removed from the UV environment and immediately applied to the teeth. Each tooth had its buccal surface in contact with the suspension for 2 h daily over a period of 21 days, simulating a home bleaching protocol. The control group utilized 16% CP (Whiteness Simple, FGM, Joinville, Brazil) as a commercial whitening gel, following the same application scheme of 2 h per day for 21 days, without undergoing the photocatalysis process. Details of the experimental groups are presented in Table 1 and illustrated in Figure 1. Each group consisted of 12 specimens (*n* = 12) throughout the experiments.**!**

### 2.3. Color Evaluation (ΔEab)

Color analysis (*n* = 12) was conducted using a Vita Easyshade spectrophotometer, assessing brightness (*L*^∗^) and color coordinates (*a*^∗^ and *b*^∗^) before and after the whitening protocols. This assessment was performed by a single calibrated operator. The *L*^∗^ value ranged from 0 (*black*) to 100 (*white*), while the *a*^∗^ and *b*^∗^ coordinates represented the shade, with *a*^∗^ measuring along the red–green axis and *b*^∗^ along the yellow–blue axis [24]. Following the calibration of the instrument, the tip was positioned parallel to the buccal surface of each tooth under consistent moisture conditions and standardized lighting. The coordinates were then recorded. The CIELAB (ΔEab) values were calculated to quantify color change. The values obtained were applied to the following formula:

 $$\begin{array}{c} \Delta E_{ab}={\left[{\left(\Delta L*\right)}^{2}+{\left(\Delta a*\right)}^{2}+{\left(\Delta b*\right)}^{2}\right]}^{1∕2} \end{array}$$

### 2.4. pH of Bleaching Gels

The pH levels of the bleaching agents (control, Exp-50%/10 min, Exp-50%/50 min, Exp-10%/10 min, Exp-10%/50 min) were assessed using pH indicator strips (Mquant, Merck, Darmstadt, Germany). Each bleaching agent was placed in an Eppendorf tube, and the pH strips were immersed in the solutions. Initial and final pH values were recorded after 2 h, with results calculated as the average of the readings for each bleaching substance.

### 2.5. Statistical Analysis

Color variation (ΔEab) results were analyzed using SigmaPlot software Version 11.0. The data were first assessed for normality using the Shapiro–Wilk test. Following confirmation of normality, a one-way ANOVA was conducted to determine if there were statistically significant differences in ΔEab among the various experimental groups. To further explore observed differences, Tukey's post hoc test was employed. A significance level of 5% was adopted to determine statistical significance. The null hypothesis for this analysis posited that there would be no significant differences in color variation between the experimental groups and the control group.

## 3. Results

### 3.1. Color Evaluation (ΔEab)

**Figure 2:**
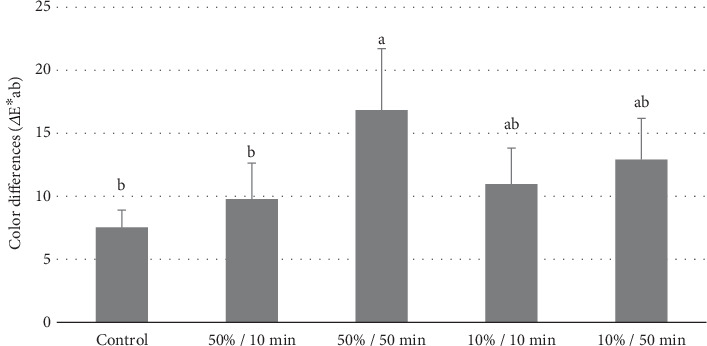
Means (standard deviation) of color change (ΔEab). The results of the statistical analysis (one-way ANOVA and Tukey's test) indicate that different small letters represent statistically significant differences (*p* < 0.05). The 50%/50 group exhibited higher values, with no statistical difference compared to the 10%/10 and 10%/50 groups, while the control and 50%/10 groups showed the lowest ΔEab.

Figure 2 presents the color analysis values, highlighting the changes observed after treatment. Among all the bleaching agents tested, the treatment with TiO₂ at a 50% concentration, activated for 50 min, resulted in the greatest color variation (ΔEab = 16.84). This treatment was statistically superior to all other groups (*p* < 0.001). The TiO₂ groups at a concentration of 10%, activated for both 10 and 50 min, achieved intermediate color difference values (ΔEab = 10.96 and ΔEab = 12.90, respectively), which did not differ significantly from each other (*p* = 0.0875). In contrast, the groups treated with 16% CP and TiO₂ at a concentration of 10%, activated for 50 min, exhibited lower color variations (ΔEab = 7.52 and ΔEab = 9.78, respectively). These two groups showed similar performance (*p* = 0.102) and were comparable to the Exp-10%/10 min and Exp-50%/10 min groups.

### 3.2. pH of Bleaching Gels

The pH of the bleaching agents—control, Exp-50%/10 min, Exp-50%/50 min, Exp-10%/10 min, and Exp-10%/50 min—remained consistent at pH 6 both before and after 2 h of the at-home bleaching protocol.

## 4. Discussion

In this pioneering study evaluating the use of TiO₂ without HP, TiO₂ was applied as a suspension to prevent interference with photocatalysis. Notably, a previous pilot study found that an Exp-10% formulation with only 2 min of UV exposure did not yield results comparable to CP. However, in the present study, the same concentration with 10 min of photocatalysis achieved bleaching effects similar to the control group, suggesting that TiO₂ could serve as a promising alternative in at-home bleaching protocols, even at lower concentrations and shorter UV exposure times. Furthermore, the Exp-50% treatment with 50 min of photocatalysis proved more effective than the control group (16% CP), leading to the rejection of the null hypothesis, which posited no significant differences between the experimental and control groups. Notably, the effectiveness of TiO₂ nanoparticles as a tooth-whitening agent without HP has not been previously documented in the literature.

The 16% CP commercial bleaching gel was utilized as a benchmark for at-home tooth whitening due to its established efficacy, demonstrating color stability for up to 42 months posttreatment, as noted in recent clinical trials [17]. While CP exhibited lower ΔEab values compared to other bleaching agents, these findings align with previously reported values (8.0–9.6) found in the literature [17]. A systematic review by De Geus et al. highlighted that at-home bleaching with 10% CP provides similar efficacy with a reduced risk and intensity of tooth sensitivity compared to higher concentrations of HP. Elevated concentrations of HP are associated with increased sensitivity and potential pulpal damage [17].

Current research in bleaching is increasingly focused on reducing HP concentrations through alternative photocatalytic agents. The use of TiO₂ nanoparticles without HP presents multiple advantages, such as cost-effectiveness and lower toxicity compared to HP [14, 15, 19, 25–28]. The photocatalytic activation of TiO₂ is achieved via heterogeneous photocatalysis in water-based suspensions, where light exposure can occur prior to application on tooth structures. This process exploits the VBs and CBs of TiO₂, with the region between them known as the “band gap.” During activation, free radicals are generated, which are essential for the bleaching mechanism [14, 18, 20, 25–28].

Recent investigations have shown that combining TiO₂ nanoparticles with HP enhances free radical formation, allowing for reduced HP concentrations (6%) when paired with 5%–10% TiO₂ [11, 14, 19, 24, 25, 27]. This combination not only improves the bleaching effect but also diminishes the reliance on higher HP concentrations, which are linked to increased toxicity and sensitivity risks [11, 12, 14, 17–19, 24, 25, 27]. Thus, TiO₂ nanoparticles demonstrate potential as a safer alternative for dental bleaching treatments, effectively minimizing adverse reactions while ensuring efficient whitening.

The application of TiO₂ at 50% concentration with 10 min of photocatalysis yielded results comparable to 16% CP, indicating its clinical effectiveness as a dental bleaching option. A key finding of this study is the importance of conducting the activation process with UV at a distance from the tooth structure, which reduces the risk of elevating pulp temperature and causing irreversible damage. The efficacy of TiO₂ at 50% concentration can be attributed to simultaneous chemical oxidation–reduction reactions occurring in the nanoparticles, generating free radicals that effectively break down large pigment molecules associated with tooth stains, such as those containing heteroatoms, carbonyl, and/or phenyl rings [14, 18, 20, 25, 26].

Achieving optimal photocatalysis necessitates delivering energy equal to or greater than the energy of the band gap, which excites electrons and facilitates the necessary oxidation–reduction reactions [14, 18, 20, 25, 26]. The photocatalysis involving TiO₂ nanoparticles is pivotal for effective dental bleaching without HP, establishing it as a promising alternative for bleaching treatments [11, 14, 17–19, 22, 24–27].

The difference in performance observed between the two activation times for TiO₂ at 50% concentration may be linked to the energy supplied during photocatalysis. While the 10-min activation period may have provided sufficient energy for initiating the oxidation-reduction reactions, higher TiO₂ concentrations increase energy demand. Thus, the 10-min activation may not have generated enough energy to fully excite the electrons, resulting in lower whitening efficiency compared to the 50-min activation period, which likely facilitated greater energy accumulation and free radical production.

In this study, UV was employed to activate the photocatalysis of TiO₂ nanoparticles. As a semiconductor, TiO₂ absorbs photons with energies at wavelengths shorter than 380 nm (UV). For the anatase phase of TiO₂, the band gap energy is approximately 3.2 eV, corresponding to a wavelength of 387 nm, while the rutile phase has a slightly lower band gap energy. The UV light used in this study, with a wavelength of 395 nm, is close to the optimal absorption range, ensuring sufficient energy for effective photocatalysis [14, 18, 20, 25, 26, 29–33].

When TiO₂ is combined with HP or other chemical agents, the positions of the conduction and VB edges are modified, enabling TiO₂ to respond to visible light, thus broadening its photocatalytic applications [24, 29–33]. Studies have demonstrated that LED curing units emitting wavelengths between 440 and 480 nm can activate TiO₂ photocatalysis when paired with HP, presenting a potential alternative for in-office bleaching, which could reduce session duration 26. However, for pure TiO₂ nanoparticles, selecting the appropriate wavelength is critical for optimal photocatalysis and free radical formation.

This investigation is the first to establish the optimal concentration and photocatalytic exposure time for TiO₂ in a peroxide-free environment. UV light activation was performed externally to avoid increasing pulp temperature. Toxicity studies indicate that bleaching gels containing TiO₂ exhibit lower cytotoxicity than those with higher HP concentrations, which may enhance whitening efficacy and reduce sensitivity in clinical settings. It is worth noting that other agents, such as bromelain, chlorine dioxide, bromelain, papain, or ficin, have also shown substantial clinical potential for developing peroxide-free gels [34, 35]. The advantage of using TiO₂ lies in its extensive prior research, particularly in dental bleaching applications.

However, the study had limitations, such as the application of the TiO₂ suspension without a custom tray and in a liquid form rather than a gel consistency, which may not be clinically feasible. Additional concentrations of TiO₂ beyond 10% and 50% were not tested, and only one wavelength of UV light was employed for photocatalysis. Furthermore, the long-term color stability postbleaching was not assessed, and the study relied on artificially stained teeth with black tea pigments, lacking pH cycling, which occurs in clinical settings and may influence bleaching effectiveness.

In conclusion, TiO₂ nanoparticles represent promising active agents for tooth bleaching, even in the absence of HP. Future studies should focus on optimizing this material's use, exploring various UV light sources and wavelengths, and evaluating changes in enamel morphology, biofilm formation, and clinical applicability in patient studies. The application of a 50 wt% TiO₂ nanoparticle suspension with 50 min of photocatalysis demonstrated significant whitening effects in bovine teeth, outperforming 16% CP in the at-home bleaching protocol.

## Figures and Tables

**Table 1:** Spreading of groups evaluated in the study.

**Group**	**Description**
Control	16% carbamide peroxide
TiO_2_ 50%/10 min	50% TiO_2_ solution with photocatalysis for 10 min
TiO_2_ 50%/50 min	50% TiO_2_ solution with photocatalysis for 50 min
TiO_2_ 10%/10 min	10% TiO_2_ solution with photocatalysis for 10 min
TiO_2_ 10%/50 min	10% TiO_2_ solution with photocatalysis for 50 min

## Data Availability

Research data can be presented upon a reasonable request.

## References

[1] Gasmi Benahmed A., Gasmi A., Menzel A. (2022). A review on natural teeth whitening. *Journal of Oral Biosciences*.

[2] Alkahtani R., Stone S., German M., Waterhouse P. (2020). A review on dental whitening. *Journal of Dentistry*.

[3] Carey C. M. (2014). Tooth whitening: what we now know. *The Journal of Evidence-Based Dental Practice*.

[4] Ubaldini A. L., Baesso M. L., Medina Neto A., Sato F., Bento A. C., Pascotto R. C. (2013). Hydrogen peroxide diffusion dynamics in dental tissues. *Journal of Dental Research*.

[5] Bersezio C., Martín J., Prieto M. V. (2019). One-year bleaching efficacy using two HP products with different pH: a double-blind randomized clinical trial. *Journal of Esthetic and Restorative Dentistry*.

[6] Pascolutti M., de Oliveira D. (2021). A radical-free approach to teeth whitening. *Dentistry Journal*.

[7] Borges A. B., de Abreu F. S., Mailart M. C., Zanatta R. F., Torres C. (2021). Efficacy and safety of bleaching gels according to application protocol. *Operative Dentistry*.

[8] Kwon S. R., Wertz P. W. (2015). Review of the mechanism of tooth whitening. *Journal of Esthetic and Restorative Dentistry*.

[9] Cavalli V., Silva B. G., Berger S. B. (2017). Effect of adhesive restoration and bleaching technique on the concentration of hydrogen peroxide in the pulp chamber. *Operative Dentistry*.

[10] Carneiro T. S., Favoreto M. W., Ferreira M. W. C. (2023). In-office dental bleaching in adolescents using 6% hydrogen peroxide with different application tips: randomized clinical trial. *Journal of Applied Oral Science*.

[11] Santana M. S., Bridi E. C., Navarro R. S. (2016). Dental bleaching with ozone: effects on color and enamel microhardness. *Acta Odontológica Latinoamericana*.

[12] Gruba A. S., Nunes G. P., Marques M. T. (2023). Influence of bleaching gels formulated with nano-sized sodium trimetaphosphate and fluoride on the physicochemical, mechanical, and morphological properties of dental enamel. *Journal of Dentistry*.

[13] Kolsuz Ozcetin H., Surmelioglu D. (2020). Effects of bleaching gel containing TiO2and chitosan on tooth surface roughness, microhardness and colour. *Australian Dental Journal*.

[14] Monteiro N. R., Basting R. T., Amaral F. L. B. D. (2020). Titanium dioxide nanotubes incorporated into bleaching agents: physicochemical characterization and enamel color change. *Journal of Applied Oral Science*.

[15] Vargas-Koudriavtsev T., Durán-Sedó R., Herrera-Sancho Ó. A. (2018). Titanium dioxide in dental enamel as a trace element and its variation with bleaching. *Journal of Clinical and Experimental Dentistry*.

[16] Antunes E. V., Basting R. T., do Amaral F. L. B. (2023). Titanium dioxide nanotubes in a hydrogen peroxide-based bleaching agent: physicochemical properties and effectiveness of dental bleaching under the influence of a poliwave led light activation. *Clinical Oral Investigations*.

[17] de Geus J. L., Wambier L. M., Boing T. F., Loguercio A. D., Reis A. (2018). At-home bleaching with 10% vs more concentrated carbamide peroxide gels: a systematic review and meta-analysis. *Operative Dentistry*.

[18] Carlos N. R., Basting R. T., Amaral F. L. B. D. (2023). Physicochemical evaluation of hydrogen peroxide bleaching gels containing titanium dioxide catalytic agent, and their influence on dental color change associated with violet LED. *Photodiagnosis and Photodynamic Therapy*.

[19] Sürmelioğlu D., Özçetin H. K., Özdemir Z. M., Yavuz S. A., Aydın U. (2021). Effectiveness and SEM-EDX analysis following bleaching with an experimental bleaching gel containing titanium dioxide and/or chitosan. *Odontology*.

[20] Hossain S. M., Tijing L., Suzuki N., Fujishima A., Kim J. H., Shon H. K. (2022). Visible light activation of photocatalysts formed from the heterojunction of sludge-generated TiO_2_ and g-CN towards NO removal. *Journal of Hazardous Materials*.

[21] Zhang X., Jin M., Liu Z. (2006). Preparation and photocatalytic wettability conversion of TiO2-based superhydrophobic surfaces. *Langmuir*.

[22] Park J. K., Kwon Y. H., Garcia-Godoy F. (2022). Teeth whitening using nitrogen doped-TiO₂ nanoparticles and hydrogen peroxide under visible light irradiation. *American Journal of Dentistry*.

[23] Panahandeh N., Mohammadkhani S., Sedighi S., Nejadkarimi S., Ghasemi A. (2023). Comparative effects of three bleaching techniques on tooth discoloration caused by tea. *Frontiers in Dentistry*.

[24] Kury M., Hiers R. D., Zhao Y. D. (2022). Novel experimental in-office bleaching gels containing co-doped titanium dioxide nanoparticles. *Nanomaterials*.

[25] Suyama Y., Otsuki M., Ogisu S. (2009). Effects of light sources and visible light-activated titanium dioxide photocatalyst on bleaching. *Dental Materials Journal*.

[26] Cuppini M., Leitune V. C. B., Souza M., Alves A. K., Samuel S. M. W., Collares F. M. (2019). In vitro evaluation of visible light-activated titanium dioxide photocatalysis for in-office dental bleaching. *Dental Materials Journal*.

[27] Eimar H., Siciliano R., Abdallah M. N. (2012). Hydrogen peroxide whitens teeth by oxidizing the organic structure. *Journal of Dentistry*.

[28] Saita M., Kobayashi K., Yoshino F. (2012). ESR investigation of ROS generated by H2O2 bleaching with TiO2 coated HAp. *Dental Materials Journal*.

[29] Setvin M., Shi X., Hulva J. (2017). Methanol on anatase TiO_2_ (101): mechanistic insights into photocatalysis. *ACS Catalysis*.

[30] Matos I. C. R. T., Kury M., de Melo P. B. G., de Souza L. V. S., Esteban Florez F. L., Cavalli V. (2023). Effects of experimental bleaching gels containing co-doped titanium dioxide and niobium pentoxide combined with violet light. *Clinical Oral Investigations*.

[31] Thacker M., Chen Y. N., Lin C. P., Lin F. H. (2021). Nitrogen-doped titanium dioxide mixed with calcium peroxide and methylcellulose for dental bleaching under visible light activation. *International Journal of Molecular Sciences*.

[32] Hurum D. C., Gray K. A., Rajh T., Thurnauer M. C. (2005). Recombination pathways in the Degussa P25 formulation of TiO2: surface versus lattice mechanisms. *The Journal of Physical Chemistry. B*.

[33] Jo W. K., Kim J. T. (2009). Application of visible-light photocatalysis with nitrogen-doped or unmodified titanium dioxide for control of indoor-level volatile organic compounds. *Journal of Hazardous Materials*.

[34] Shakeel M., Jabeen F., Shabbir S., Asghar M. S., Khan M. S., Chaudhry A. S. (2016). Toxicity of nano-titanium dioxide (TiO_2_-NP) Through various routes of exposure: a review. *Biological Trace Element Research*.

[35] Ribeiro J. S., de Oliveira da Rosa W. L., da Silva A. F., Piva E., Lund R. G. (2020). Efficacy of natural, peroxide-free tooth-bleaching agents: a systematic review, meta-analysis, and technological prospecting. *Phytotherapy Research*.

